# Nanoencapsulation of a Far-Red Absorbing Phthalocyanine into Poly(benzylmalate) Biopolymers and Modulation of Their Photodynamic Efficiency

**DOI:** 10.1021/acs.biomac.3c01382

**Published:** 2024-05-16

**Authors:** Zeynel Şahin, Emel Önal, Lamiaa M. A. Ali, Denis Durand, Atefeh Emami, Marie Touré, Umit İşci, Magali Gary-Bobo, Sandrine Cammas-Marion, Fabienne Dumoulin

**Affiliations:** † Faculty of Technology, Department of Metallurgical & Materials Engineering, Marmara University, 34722 Istanbul, Türkiye; ‡ Faculty of Engineering, Doğuş University, Ümraniye, 34775 Istanbul, Türkiye; § IBMM, Univ Montpellier, CNRS, ENSCM, 34093 Montpellier, France; ∥ Department of Biochemistry Medical Research Institute, University of Alexandria, 21561 Alexandria, Egypt; ⊥ Faculty of Engineering and Natural Sciences, Biomedical Engineering Department, Acıbadem Mehmet Ali Aydınlar University, Ataşehir, 34752 Istanbul, Türkiye; # Univ Rennes, ENSCR, INSA Rennes, CNRS, ISCR (Institut des Sciences Chimiques de Rennes)—UMR 6226, F-35000 Rennes, France; ¶ INSERM, INRAE, Univ Rennes, Institut NUMECAN (Nutrition Metabolisms and Cancer), U1317, F-35000 Rennes, France

## Abstract

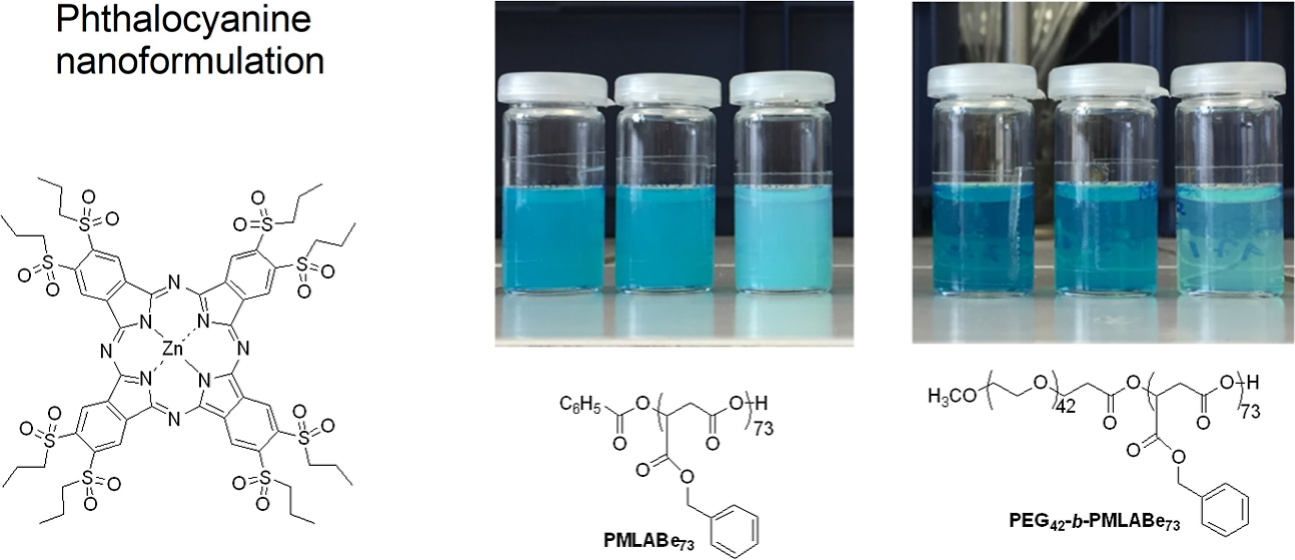

Two different poly(benzylmalate) biopolymers, a hydrophobic non-PEGylated (**PMLABe**_**73**_) and an amphiphilic PEGylated derivative (**PEG**_**42**_**-*b*-PMLABe**_**73**_), have been used to encapsulate a phthalocyanine chosen for its substitution pattern that is highly suitable for photodynamic therapy. Different phthalocyanine/(co)polymers ratios have been used for the nanoprecipitation. A set of six nanoparticles has been obtained. If the amphiphilic PEGylated copolymer proved to be slightly more efficient for the encapsulation and to lower the aggregation of the phthalocyanine inside the nanoparticles, it is, however, the hydrophobic **PMLABe**_**73**_-based nanoparticles that exhibited the best photodynamic efficiency.

## 1 Introduction

Photodynamic therapy (PDT) is now well-known as a valuable alternative cancer treatment, with many clinical trials and approved indications.^1^ PDT has numerous advantages over the three most common treatments: surgery, chemo, and radio therapies, such as limited side effects and better patient quality of life during and after the treatment, not mentioning that it has saved many lives when all other approved options remained unsuccessful.^2^ Yet drawbacks remain, such as photosensitizers’ limited biocompatibility, limited light tissue penetration, hypoxia,^3^ improvable tumor-specific selective accumulation,^4^ and days-lasting post-treatment residual photosensitivity. Many strategies are developed to enhance photodynamic efficiency,^5^ such as the use of photosensitizers excitable with far-red or NIR wavelength to avoid exciting hence damaging endogenous chromophores and also because these wavelengths penetrate more deeply into biological tissues. In this respect, phthalocyanines are chosen photosensitizers.^6^ The photoproperties of phthalocyanines can be modulated by modifying their metalation and substitution pattern.^7^ Their biocompatibility is another issue: unsubstituted Zn phthalocyanine is hardly soluble in all solvents, but its liposomal formulation gave promising clinical results.^8^ Phthalocyanines can be made water-soluble^9^ or formulated to be administrated. Nanoformulation has the additional advantage to likely benefit from the enhanced permeation and retention (EPR) effect.^10^ Covalent grafting of phthalocyanines onto biocompatible polymers^11^ or nanoparticles^12^ has been reported. Encapsulation into various carriers is a more flexible technique.^13^ Polyvinylpyrrolidone,^14^ Pluronics poloxamers,^15^ among others, have been used to encapsulate nonwater-soluble phthalocyanines and gave excellent photodynamic outcome.

Poly(β-malic acid) (PMLA), which is naturally found in apples and in grapes, is a biocompatible, nontoxic, nonimmunogenic, and water-soluble polyester, having the significant advantage of being metabolized into malic acid, an intermediate in the mammalian tricarboxylic acid cycle (also known as the citric acid cycle, the Krebs cycle, or the Szent–Györgyi–Krebs cycle), and completely biodegraded.^16^ It has already been used in various biomedical applications and more especially for drug delivery.^17^ Poly(benzyl malate) (PMLABe) polymers are a subclass of PMLA polymers that also obtained by ring opening polymerization (ROP)^18^ and that have been used for the encapsulation of various drugs, such as nickel–bis(dithiolene) complexes,^19^ porphyrins,^20^ and chemotherapeutic drugs such as doxorubicin.^21^ Their PEGylated copolymeric derivatives have also been employed to encapsulate photothermal metal–bis(dithiolene) complexes^22^ or a lipophilic radiotracer.^23^

In order to explore the relevance of using PMLABe polymers for the encapsulation and delivery of hydrophobic phthalocyanines for PDT, a phthalocyanine with an octaalkylsulfonyl substitution pattern (**ZnPc(SO**_**2**_**Prop)8**) known to have good singlet oxygen generation properties has been selected and encapsulated into two different PMLABe polymers. In addition, different encapsulation conditions and ratios have been tested to evaluate the effect on the photodynamic outcome.

## 2 Experimental Part

### 2.1 Materials and Methods

All chemicals were used as received. 4,5-Bis(propylsulfonyl)phthalonitrile was prepared as previously described.^24^ α-Methoxy-ω-carboxylic acid PEG_42_ (*M*_w_ = 2015 g/mol, *n* = 42) were purchased from PEG Iris Biotech. Disposable PD-10 Desalting Column, with Sephadex G-25 resin for 1.0–2.5 mL samples—Cytiva were used to isolate the nanoparticles. Dynamic light scattering (DLS) measurements are performed on a Nanosizer ZS90 (Malvern) at 25 °C, with a He–Ne laser at 633 nm and a detection angle of 90 °C. UV–visible measurements for the determination of the encapsulation efficiency and the calibration curve were performed on a V-750 UV–visible spectrophotometer (JASCO) from 400 to 800 nm, and the solutions’ absorbance was measured at 686 nm. All the materials and methods regarding the UV–visible and fluorescence characterization of the nanoparticles are detailed in the Supporting Information.

### 2.2 Synthesis

#### 2.2.1 Synthesis of **PMLABe**_**73**_ and **PEG**_**42**_**-*b*-PMLABe**_**73**_ Polymers

**PMLABe**_**73**_ was synthesized by anionic ring opening polymerization (aROP) of benzyl malolactonate (MLABe) in the presence of tetraethylammonium benzoate (C_6_H_5_COO^–+^NEt_4_) as initiator as previously described.^25^**PEG**_**42**_**-*b*-PMLABe**_**73**_ was synthesized using a slightly modified method described previously.^26^ Briefly, MLABe was polymerized by aROP method using tetraethylammonium salts of α-methoxy-ω-carboxylate-PEG_42_ (PEG_42_) obtained by the reaction between 1 equiv of tetraethylammonium hydroxide and 1 equiv of α-methoxy-ω-carboxylic acid PEG_42_. For both polymers, the molar mass of PMLABe was fixed at 15,000 g/mol by the MLABe/initiator ratio MLABe/initiator (73/1). The hydrophobic homopolymer (**PMLABe**_**73**_) and the amphiphilic block copolymer (**PEG**_**42**_**-*b*-PMLABe**_**73**_) were purified by precipitation and characterized by proton nuclear magnetic resonance (^1^H NMR) and size-exclusion chromatography (SEC) (Figures S1 and S4).

#### 2.2.2 Synthesis of **ZnPc(SO**_**2**_**Prop)8**

4,5-Bis(propylsulfonyl)phthalonitrile^24^ (400 mg, 1.17 mmol) and Zn(OAc)_2_ (107 mg, 0.58 mmol) were stirred overnight at 140 °C in a mixture of o-dichlorobenzene-DMF (3:1) under argon. The solvent was then removed under reduced pressure. **ZnPc(SO**_**2**_**Prop)8** was isolated by chromatography on silica gel using a mixture of dichloromethane/ethanol (100/1). Yield: 18% (75 mg). ATR-FT-IR (ν, cm^–1^): 2968, 2934, 2878, 1606, 1566, 1484, 1456, 1403, 1287, 1137, 1079, 941, 922, 746, 714, 644, 523. ^1^H NMR (500 MHz, CDCl_3_) δ, ppm: 10.33 (8 H, br s, aromatics), 3.89 (16 H, br s, SCH_2_), 2.04 (16 H, m, S CH_2_CH_2_), 1.09 (24 H, m, CH_3_). MALDI-TOF-MS (DHB) *m*/*z*: 1427.126 [M]^+^; calcd for C_56_H_64_N_8_O_16_S_8_Zn, 1427.028.

### 2.3 Preparation and Characterization of **ZnPc(SO**_**2**_**Prop)8**-Loaded **PMLABe**_**73**_ and **PEG**_**42**_**-*b*-PMLABe**_**73**_-Based Nanoparticles

#### 2.3.1 Protocols for the Preparation of **ZnPc(SO**_**2**_**Prop)8**-Loaded Nanoparticles

A stock solution of **ZnPc(SO**_**2**_**Prop)8** in tetrahydrofuran was first prepared at a concentration of 1 mg/mL. **ZnPc(SO**_**2**_**Prop)8**-loaded nanoparticles were prepared as follows: 5 mg of polymer (**PMLABe**_**73**_ or **PEG**_**42**_**-*b*-PMLABe**_**73**_) was weighted and solubilized in a defined volume of THF, followed by the addition of the adequate volume of **ZnPc(SO**_**2**_**Prop)8** solution, the total volume of THF being 1 mL (Table 1). This blue solution was quickly added to 2 mL of water with vigorous stirring. The milky-blue mixture was stirred at room temperature for 10 min. THF was then evaporated under a vacuum on a rotary evaporator (the vacuum was lowered to 80 mbar and maintained for 10 min). For all of the samples, the presence of a few traces of precipitate glued on the flask walls is noted after the evaporation of the THF. The volume of the final solution was adjusted to 2 mL by adding the necessary amount of water. The solution was then loaded onto a Sephadex column. Once the sample had entered the column, 0.5 mL of water was added. When all the water had entered the column, 3.5 mL of water was added. No trace of free Pc was visible on the column. The nanoparticles loaded with **ZnPc(SO**_**2**_**Prop)8** were recovered in a vial (final volume ≈ 3.5 mL) and the resulting suspension was analyzed by DLS (Table 2).

**Table 1:** Volume of THF Used to Solubilize the Polymer and Volume of **ZnPc(SO**_**2**_**Prop)8** THF Solution Added

	volume of THF added to solubilize the polymer (mL)	volume of **ZnPc(SO**_**2**_**Prop)8** solution in THF (mL)
NPs loaded with 10%Pc	500	500
NPs loaded with 5%Pc	750	250
NPs loaded with 1%Pc	950	50

**Table 2:** Characteristics of the **PMLABe**_**73**_**[Pc]** and **PEG**_**42**_**-*b*-PMLABe**_**73**_**[Pc]** Nanoparticles^a^

	before concentration		after concentration	
	*D*_h_ (nm)	PDI	*D*_h_ (nm)	PDI
**PMLABe**_**73**_**[Pc10%]**	103	0.22	97	0.21
**PMLABe**_**73**_**[Pc5%]**	110	0.22	102	0.22
**PMLABe**_**73**_**[Pc1%]**	136	0.15	130	0.14
**PEG**_**42**_**-*b*-PMLABe**_**73**_**[Pc10%]**	60	0.36	55	0.43
**PEG**_**42**_**-*b*-PMLABe**_**73**_**[Pc5%]**	82	0.29	69	0.32
**PEG**_**42**_**-*b*-PMLABe**_**73**_**[Pc1%]**	91	0.19	81	0.18

a The *D*_h_ (intensity mean) and PDI were measured by DLS.

#### 2.3.2 Concentration of Nanoparticles Suspensions for In Vitro Tests

In order to obtain a polymer concentration of 5 mg/mL, the nanoparticles’ suspensions were ultracentrifuged/filtered on microcon systems (MWCO membrane = 10 kDa). The nanoparticle suspensions were therefore placed in the filters of the microcons, the system was then centrifuged at 15,000*g* for 7 min. Then, the filters were inverted and centrifuged at 1000*g* for 1 min. The recovered suspensions were then diluted in the appropriate volume of water to obtain a total volume of 1 mL, i.e., a polymer concentration of 5 mg/mL. The suspensions were analyzed by DLS (Table 2) and UV spectroscopy (Table 4).

#### 2.3.3 Determination of the Encapsulation Efficiency by UV–Vis Spectroscopy

600 μL of each nanoparticles suspension (5 mg/mL) loaded with **ZnPc(SO**_**2**_**Prop)8** were used for the in vitro tests. The remaining 400 μL of nanoparticles suspension were used to perform UV analyses to determine the encapsulation rates. For that, 400 μL of water were added to each nanoparticles’ suspension: the theoretical concentrations of **ZnPc(SO**_**2**_**Prop)8** were thus the following: for **PMLABe**_**73**_**[Pc10%]** and **PEG**_**42**_**-*b*-PMLABe**_**73**_**[Pc10%]**: 250 μg/mL, for **PMLABe**_**73**_**[Pc5%]** and **PEG**_**42**_**-*b*-PMLABe**_**73**_**[Pc5%]**: 125 μg/mL, and for **PMLABe**_**73**_**[Pc1%]** and **PEG**_**42**_**-*b*-PMLABe**_**73**_**[Pc1%]**: 25 μg/mL. 100 μL of each nanoparticles’ diluted suspension were added to 900 μL of THF, the THF/water ratio was therefore 90/10 (dilution = 10). 250 μL of the two nanoparticles’ suspensions containing 10 wt % of **ZnPc(SO**_**2**_**Prop)8** were diluted in 750 μL of THF/water 90/10 solution (dilution = 4); 500 μL of the two nanoparticles’ suspensions containing 5 wt % of **ZnPc(SO**_**2**_**Prop)8** were diluted in 500 μL of THF/water 90/10 solution (dilution = 2); and the two nanoparticles’ suspensions containing 1 wt % of **ZnPc(SO**_**2**_**Prop)8** were not diluted. The UV spectrum of each nanoparticles’ suspension were recorded between 400 and 800 nm, and the absorbance of each sample was measured at 686 nm. The absorbance of each sample allowed quantification of the **ZnPc(SO**_**2**_**Prop)8** concentration and consequently the encapsulation efficiency (E.E. %) of each formulation (Table 3).

**Table 3:** Concentration in **ZnPc(SO**_**2**_**Prop)8** Measured by UV–Vis and Encapsulation Efficiencies (E.E.)

	[Pc]_initial_ (μg/mL)	[Pc]_meas_ (μg/mL)	E.E. %	[Pc]_meas_ (μM)
**PMLABe**_**73**_**[Pc10%]**	250	100	40	70.1
**PMLABe**_**73**_**[Pc5%]**	125	38	30	26.6
**PMLABe**_**73**_**[Pc1%]**	25	14	57	9.8
**PEG**_**42**_**-*b*-PMLABe**_**73**_**[Pc10%]**	250	108	43	75.7
**PEG**_**42**_**-*b*-PMLABe**_**73**_**[Pc5%]**	125	69	55	48.4
**PEG**_**42**_**-*b*-PMLABe**_**73**_**[Pc1%]**	25	19	76	13.3

#### 2.3.4 Calibration Curve

Standard solutions of **ZnPc(SO**_**2**_**Prop)8** in THF/water 90/10 were prepared with concentration ranging from 0.4688 to 15 μg/mL. Their UV spectra were recorded between 400 and 800 nm and the absorbance of each standard solution was measured at 686 nm, allowing us to draw a calibration curve (Figure S9).

### 2.4 Photodynamic Therapy

#### 2.4.1 Cell Culture

Human breast adenocarcinoma cell line (MCF-7) was maintained in Dulbecco’s modified Eagle’s medium (DMEM/F12) supplemented with 10% fetal bovine serum and 1% penicillin/streptomycin. Cells were allowed to grow in a humidified atmosphere at 37 °C under 5% CO_2_.

#### 2.4.2 In Vitro Dark Toxicity Studies

MCF-7 cells were seeded in a 96-well plate at a density of 5000 cells per well. After 24 h of cell growth, cells were treated with different concentrations of PEGylated and non-PEGylated Pc-loaded nanoparticles (from 0 to 300 μg mL^–1^) for 72 h. Cytotoxicity was evaluated using a 4,5-dimethylthiazol-2-yl)-2,5-diphenyltetrazolium bromide (MTT) assay. Briefly, cells were incubated for 4 h with 0.5 mg mL^–1^ of MTT in media. The MTT/media solution was then removed, and the precipitated formazan crystals were dissolved in equal volume solution of ethanol/DMSO. After 20 min of shaking, the solution optical density (OD) was read at 540 nm using microplate reader. The OD values are directly correlated with the number of living cells in the well. Cell viability was calculated as % viability = OD of treated cell/OD of vehicle control × 100.

#### 2.4.3 Photodynamic Therapy Experiment

MCF-7 cells were seeded in 96-well plate. Twenty-four h after, cells were treated with 50 μg mL^–1^ of PEGylated and non-PEGylated Pc-loaded nanoparticles for 24 h. Cells treated with the vehicle were considered as a control. After the incubation time, cells were exposed or not to laser beam at 650 nm for 20 min (11.25 J cm^–2^). Two days after irradiation, the phototoxicity effect of nanoparticles was assessed using the MTT assay as previously described.

#### 2.4.4 Cellular Reactive Oxygen Species Detection

MCF-7 cells were seeded in a 96-well plate. Twenty-four h after, cells were treated with 50 μg mL^–1^ of **PMLABe**_**73**_**[Pc1%]** and **PEG**_**42**_**-PMLABe**_**73**_**[Pc1%]** nanoparticles for 24 h. For reactive oxygen species (ROS) detection, a 2′,7′-dichlorodihydrofluorescein diacetate (DCFDA) cellular ROS detection assay kit (Abcam) was used. DCFDA is a fluorogenic dye that undergoes intracellular deacetylation to a nonfluorescent compound, which is oxidized by ROS to form fluorescent 2′,7′-dichlorofluorescein (DCF). Briefly, nanoparticles-treated cells and control cells were incubated with or without 20 μM of DCFDA for 45 min at 37 °C; then, cells were exposed or not to irradiation at 650 nm for 20 min (11.25 J cm^–2^). After irradiation, cells were washed twice and then visualized using a Leica DM.IRB microscope; green fluorescence was excited at 485 nm. Green fluorescence shows the ROS production, which is a consequence of the photodynamic effect.

## 3 Results and Discussion

### 3.1 Choice of Phthalocyanine

**Figure 1:**
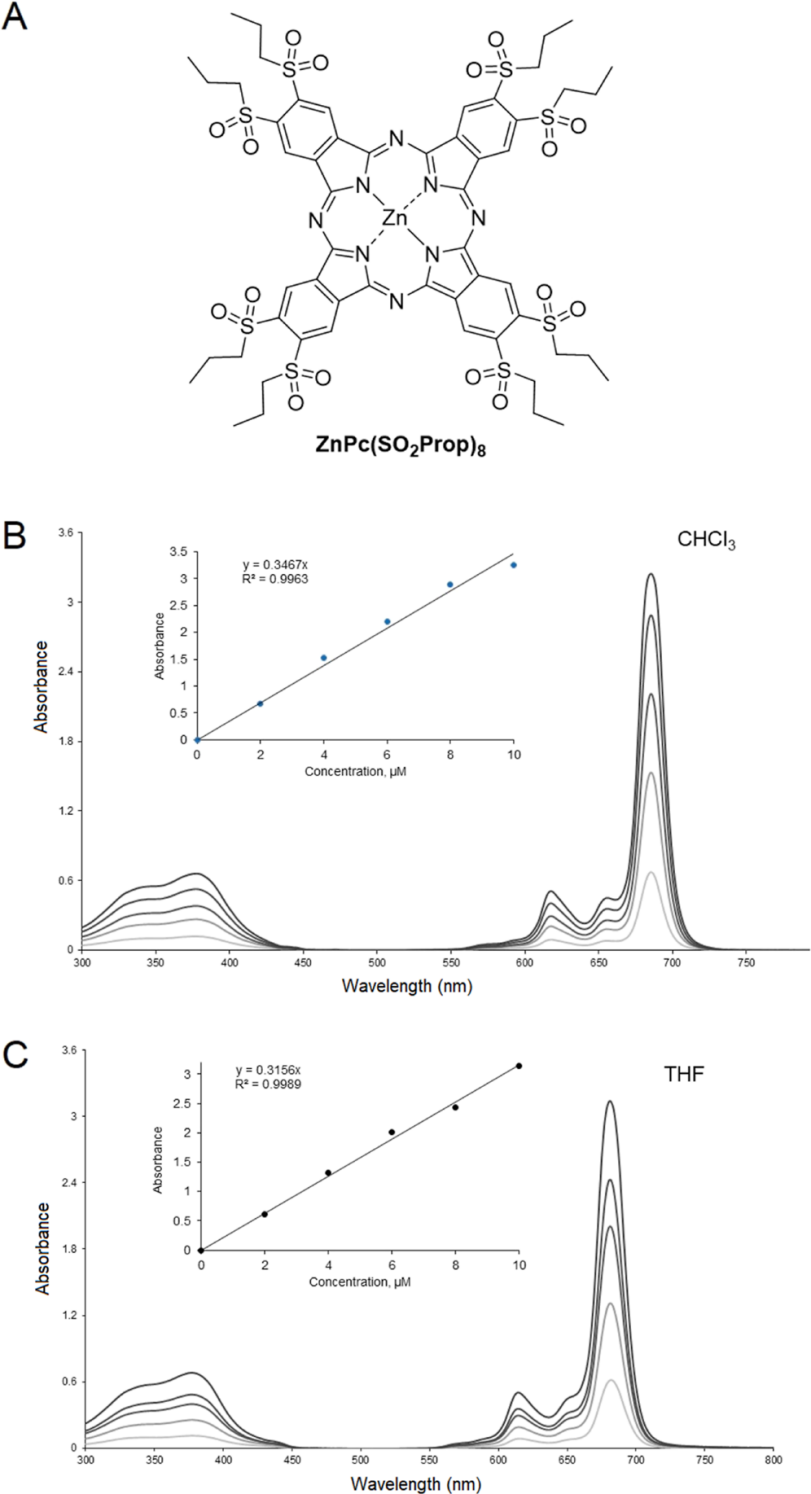
A) Structure of **ZnPc(SO**_**2**_**Prop)8**. (B) UV–vis spectra were recorded in chloroform (2–10 μM). (C) UV–vis spectra in THF (2–10 μM).

**ZnPc(SO**_**2**_**Prop)8** (Figure 1) was selected because it exists under a single isomer, which is good for potential further clinical transition, and because the octa alkylsulfonyl substitution pattern is known to induce good singlet oxygen generation, as previously measured on the analogous octa hexylsulfonyl Zn phthalocyanine.^7a^**ZnPc(SO**_**2**_**Prop)8** used for this work has been reported in the literature as a byproduct of the synthesis of asymmetrically substituted phthalocyanines.^24,27^ Its UV–vis spectra in chloroform and THF (Figure 1) showed that the chain length (propyl vs hexyl) does not affect the photoproperties and that the previously reported one^7a^ can be used as a reference.

### 3.2 Preparation and Characterization of **ZnPc(SO**_**2**_**Prop)8**-Loaded **PMLABe**_**73**_ and **PEG**_**42**_**-*b*-PMLABe**_**73**_-Based Nanoparticles

**Scheme 1:**
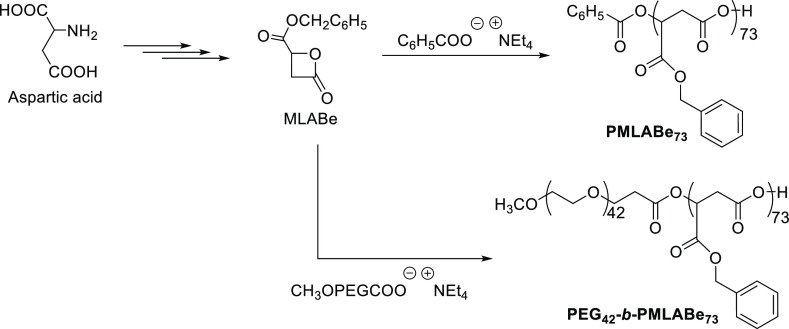
Synthetic Route to **PMLABe**_**73**_ and **PEG**_**42**_**-*b*-PMLABe**_**73**_ Polymeric Materials

Polymeric materials **PMLABe**_**73**_ and **PEG**_**42**_**-*b*-PMLABe**_**73**_ were first synthesized by aROP of benzyl malolactonate (MLABe), prepared in four steps from aspartic acid,^25^ using tetraethylammonium benzoate and tetraethylammonium salts of α-methoxy-ω-carboxylate PEG_42_,^26,28^ respectively, as initiators (Scheme 1). Both the synthesis of the monomer (MLABe) and its (co)polymerization are well-mastered and lead to reproducible results in terms of polymers’ structures and physicochemical properties. Moreover, the molar mass of the PMLABe, for both the homopolymer and the block copolymer, is determined by the ratio monomer/initiator, and fixed, for the present study, at 15,000 g/mol, i.e., a ratio monomer/initiator of 73/1.

^1^H NMR spectrum (to confirm the structure) and SEC (to determine the molar masses and dispersity) analyses were in good agreement with both the expected structures and molar masses for both the hydrophobic **PMLABe**_**73**_ and the amphiphilic block copolymer **PEG**_**42**_**-*b*-PMLABe**_**73**_.^26,28a^

The well-defined hydrophobic homopolymer (**PMLABe**_**73**_) and the amphiphilic block copolymer (**PEG**_**42**_**-*b*-PMLABe**_**73**_) were then used to prepare **ZnPc(SO**_**2**_**Prop)8**-loaded nanoparticles using nanoprecipitation,^29^ previously described to prepare PMLABe-based nanoparticles loaded or not with a hydrophobic molecule of interest.^26,28^ Three amounts of **ZnPc(SO**_**2**_**Prop)8** were encapsulated into both type of nanoparticles: 10, 5, and 1 wt % relative to the mass of (co)polymer. Nanoprecipitation is a simple and reproducible technique that consists of the rapid addition of an organic solution to an aqueous phase under vigorous stirring. The organic phase is usually prepared from a solvent miscible with water (such as THF or acetone), a solvent that contains the polymeric materials and the hydrophobic molecules of interest to be encapsulated. The good solubility of **ZnPc(SO**_**2**_**Prop)8** in THF prompted to choice of this solvent for the encapsulation procedure. The presence of **ZnPc(SO**_**2**_**Prop)8** had no significant influence on NPs formation at the studied concentrations, since well-defined NPs have been obtained. Upon the addition to the aqueous solution, and as a result of their structure, the polymeric materials spontaneously aggregate while the hydrophobic molecules of interest are entrapped into the hydrophobic core of the formed nanoparticles. Once the organic solvent is removed under vacuum, a stable nanoparticles’ suspension is thus obtained. While hydrophobic homopolymers lead to simple nanoparticles, amphiphilic block copolymers lead to nanoobjects with a core–shell structure. Usually, it is observed that nanoparticles constituted by hydrophobic homopolymers have slightly higher hydrodynamic diameters than the core–shell ones obtained from amphiphilic block copolymers.^26^

**Figure 2:**
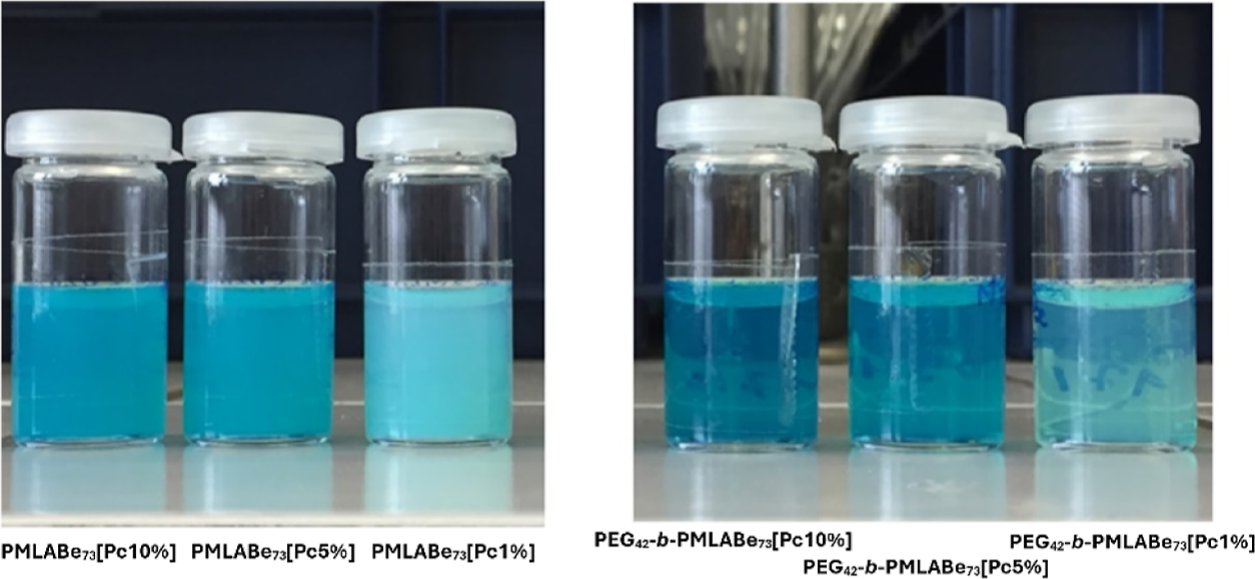
Aspect of the **ZnPc(SO**_**2**_**Prop)8**-loaded nanoparticles (fractions collected from Sephadex).

Nanoprecipitation has been achieved from six different THF solutions containing either hydrophobic homopolymer (**PMLABe**_**73**_) and the amphiphilic block copolymer (**PEG**_**42**_**-*b*-PMLABe**_**73**_), and initial contents of **ZnPc(SO**_**2**_**Prop)8** varying from 10 to 1 wt % relative to the (co)polymer mass. After the elimination of the nonencapsulated **ZnPc(SO**_**2**_**Prop)8** by filtration through a Sephadex G25 column, the resulting suspensions were obtained (Figure 2).

The nanoparticles were first analyzed by DLS to determine their hydrodynamic diameters (*D*_h_) and polydispersities. The DLS measurements were performed directly on the obtained suspensions without any dilution (Table 2). As expected,^26^ the hydrodynamic diameters of **PMLABe**_**73**_-based nanoparticles are higher than those observed for **PEG**_**42**_**-*b*-PMLABe**_**73**_-based nanoparticles. Moreover, higher initial contents of **ZnPc(SO**_**2**_**Prop)8** induce higher samples’ dispersities. Suspensions of **ZnPc(SO**_**2**_**Prop)8**-loaded **PEG**_**42**_**-*b*-PMLABe**_**73**_ nanoparticles are more limpid than **PMLABe**_**73**_ ones, much more “milky”, because the **PEG**_**42**_**-*b*-PMLABe**_**73**_ nanoparticles are smaller. The nanoparticles’ suspensions were then concentrated to reach polymers’ concentrations compatible with in vitro assays. To this end and as described in the Experimental Part, all the nanoparticles’ suspensions were ultracentrifugated/filtrated through Micro-Con devices and correctly diluted to reach (co)polymers’ concentration of 5 mg/mL. The resulting nanoparticles’ suspensions were again analyzed by DLS to check whether this protocol altered their hydrodynamic diameters and dispersities (Table 2). The comparison of the values contained in Table 2 highlights that the ultracentrifugation/filtration treatment slightly affected the hydrodynamic diameters and the dispersities of the resulting nanoparticles’ suspensions.

The encapsulation efficiency (E.E. %) for the nanoparticles was then evaluated by UV–vis measurements. First, a calibration curve using **ZnPc(SO**_**2**_**Prop)8** at different concentrations in a 90/10 vol % solution of THF/water was obtained (Figure S8A). Next, 100 μL of the 5 mg/mL suspensions were added to 900 μL of THF, the resulting THF/water ratio being therefore 90/10 as for the calibration curve. The THF/water solutions containing both the (co)polymers under the nonaggregated form and the free Pc were analyzed by UV–vis to measure the absorbance at 686 nm. Thanks to the previously established calibration curve (Figure S8B), the real contents in **ZnPc(SO**_**2**_**Prop)8** of each nanoparticles’ suspensions were determined (Table 3). The encapsulation efficiencies (E.E. %) were calculated as follows
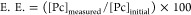


As shown by the results gathered in Table 3, the encapsulation efficiencies are much higher for the lower initial amounts of phthalocyanine (1 wt %). Such results agree with the experimental observation. Indeed during the formulation of nanoparticles with the higher amounts of Pc (10 and 5 wt %), we have observed, after evaporation of the THF, the formation of some blue precipitate corresponding to nonencapsulated **ZnPc(SO**_**2**_**Prop)8**, while with the lowest amounts of **ZnPc(SO**_**2**_**Prop)8** (1 wt %), we did not observe precipitation after THF removal. It appears also that **PEG**_**42**_**-*b*-PMLABe**_**73**_ polymers are slightly more efficient to encapsulate **ZnPc(SO**_**2**_**Prop)8** during the nanoprecipitation process than non-PEGylated **PMLABe**_**73**_ polymers.

**Figure 3:**
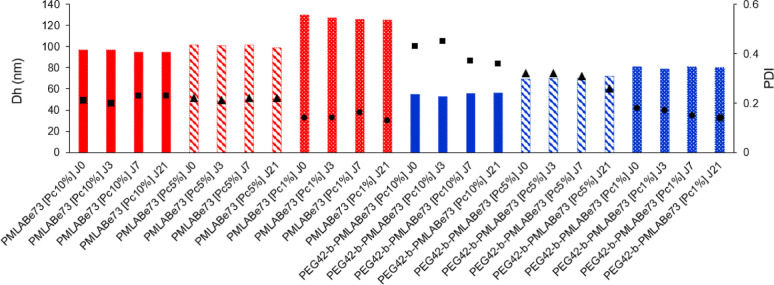
Evolution of the hydrodynamic diameter (bars in red for **PMLABe**_**73**_ and in blue for **PEG**_**42**_**-*b*-PMLABe**_**73**_ materials) and of the dispersities (black symbols) of the nanoparticles over time upon storage at 4 °C. Values are average of triplicate measurements.

Finally, the stability at 4 °C of the nanoparticles’ suspensions was followed by DLS (Table 4 and Figure 3). The results demonstrate the very good stability of the nanoparticles’ suspensions upon storage at 4 °C, as highlighted by the stability of both hydrodynamic diameters and polydispersities.

**Table 4:** Evolution of the Hydrodynamic Diameter (*D*_h_) and the Polydispersity (PDI) of Nanoparticles’ Suspensions upon Storage at 4 °C

	day 0	day 3	day 7	day 21
	*D*_h_ (nm)/PDI	*D*_h_ (nm)/PDI	*D*_h_ (nm)/PDI	*D*_h_ (nm)/PDI
**PMLABe**_**73**_**[Pc10%]**	97/0.21	97/0.20	95/0.23	95/0.23
**PMLABe**_**73**_**[Pc5%]**	102/0.22	101/0.21	102/0.22	99/0.22
**PMLABe**_**73**_**[Pc1%]**	130/0.14	127/0.14	126/0.16	125/0.13
**PEG**_**42**_**-*b*-PMLABe**_**73**_**[Pc10%]**	55/0.43	53/0.45	56/0.37	56/0.36
**PEG**_**42**_**-*b*-PMLABe**_**73**_**[Pc5%]**	69/0.32	70/0.32	70/0.31	72/0.26
**PEG**_**42**_**-*b*-PMLABe**_**73**_**[Pc1%]**	81/0.18	79/0.17	81/0.15	80/0.14

**Figure 4:**
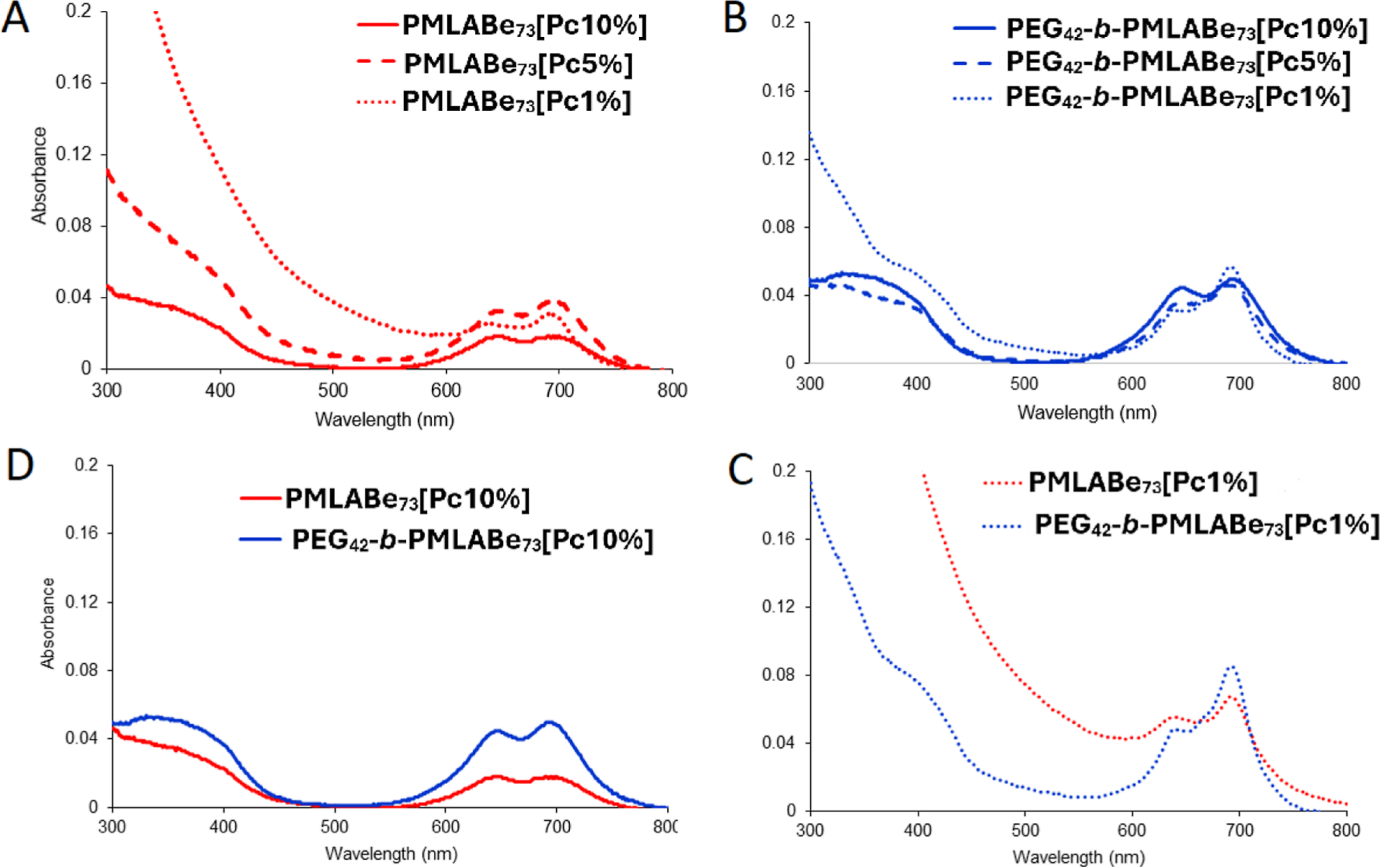
UV–vis spectra of the nanoparticles in water. **ZnPc(SO**_**2**_**Prop)8** loading (1, 5, and 10 wt %) in **PMLABe**_**73**_ (A) and in **PEG**_**42**_**-*b*-PMLABe**_**73**_ (B). Different polymeric materials and same **ZnPc(SO**_**2**_**Prop)8** loading: 1 (C) and 10 wt % (D).

The UV–vis spectra of all nanoparticles in water have been recorded (Figures 4 and S9). All of the phthalocyanines appear to be aggregated inside the nanoparticles, regardless of the loading ratio and of the polymer used to prepare the nanoparticles. To study the effect of the different loading ratios on the phthalocyanine aggregation state for both polymeric materials, UV–vis spectra in which the phthalocyanine has the same concentration (established for each nanoparticle depending on the E.E.) have been recorded. Aggregation is more pronounced in **PMLABe**_**73**_ nanoparticles, especially for the 10 wt % loading (Figure 4A), whereas in **PEG**_**42**_**-*b*-PMLABe**_**73**_ nanoparticles, the aggregation is overall less marked for a 10 wt % loading and the less split and less flattened Q-band for the 1 wt % loading indicates that **ZnPc(SO**_**2**_**Prop)8** is more monomerized, however not enough to observe fluorescence as it is much more sensitive to aggregation. These observations make sense as more phthalocyanines are introduced in the same amount of polymer, leading to their local aggregation inside the nanoparticle and the counterintuitive fact that more loaded nanoparticles have lower absorption. The fact that, compared to **PMLABe**_**73**_ nanoparticles, **PEG**_**42**_**-*b*-PMLABe**_**73**_ nanoparticles slightly limit the aggregation of the phthalocyanine is confirmed when looking at the superimposed UV–vis spectra of the phthalocyanine at the same concentration and same loading (1 wt % in Figures 4C and 10 wt % in Figure 4D) in both type of nanoparticles.

Fluorescence is even more sensitive to aggregation than is electronic UV–vis absorption. Due to their aggregated state inside the nanoparticles, no fluorescence could be observed from their aqueous solutions. However, each nanoparticle was disrupted by being diluted with THF reaching a final THF 9/water 1 ratio, and their fluorescence spectrum was measured. The fluorescence was perfectly restored (Figure S10), showing that the quenching of the photoproperties is only due to the encapsulation and is reversible.

### 3.3 Biological Studies

**Figure 5:**
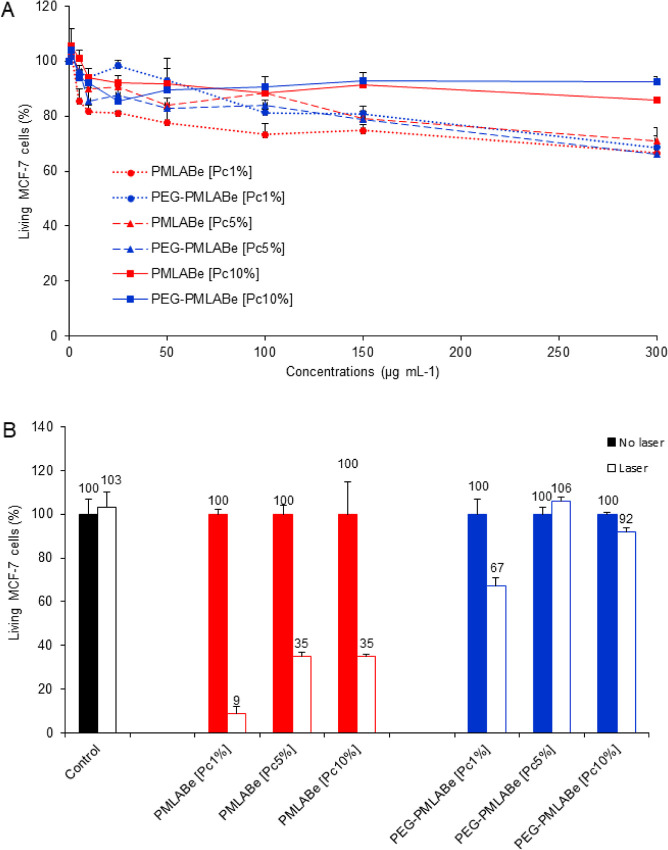
A) Cytotoxicity study of MCF-7 cells incubated with different concentrations of PEGylated and non-PEGylated **ZnPc(SO**_**2**_**Prop)8**-loaded nanoparticles for 72 h. (B) PDT effect of PEGylated and non-PEGylated **ZnPc(SO**_**2**_**Prop)8**-loaded nanoparticles on MCF-7 cells incubated with 50 μg mL^–1^ for 24 h. Cells were irradiated with a continuous laser for 20 min at 650 nm (11.25 J cm^–2^). Data are presented as (mean ± SEM), *n* = 3.

First, the cytotoxic effect of all nanoparticles was evaluated on MCF-7 cells. All nanoparticles were incubated with increasing amounts of nanoparticles and exhibited low cytotoxic effect, which was more obvious for **PMLABe**_**73**_**[Pc10%]** and **PEG**_**42**_**-*b*-PMLABe**_**73**_**[Pc10%]** nanoparticles as 14 and 7%, respectively, of cell death was detected at 300 μg mL^–1^. Other nanoparticles at 300 μg mL^–1^ showed that cell death ranged between 29 and 34% (Figure 5A). The concentration of 50 μg mL^–1^ was therefore considered adequate, in terms of cytotoxicity, for the subsequent experiments. It corresponds to a safety concentration, for which no significant cell death and no significant differences were observed between all the formulations.

The photodynamic efficiency of **ZnPc(SO**_**2**_**Prop)8**-loaded non-PEGylated **PMLABe**_**73**_ and of PEGylated **PEG**_**42**_**-*b*-PMLABe**_**73**_ nanoparticles on MCF-7 cells was studied, as shown in Figure 5B. Cells were treated with 50 μg mL^–1^ of nanoparticles for 24 h and then exposed or not to laser irradiation at 650 nm for 20 min (11.25 J cm^–2^). 91% of cell death was detected in cells treated with **PMLABe**_**73**_**[Pc1%]** (Figure 5B), while for both **PMLABe**_**73**_**[Pc5%]** and **PMLABe**_**73**_**[Pc10%]**, 65% of cell death were detected. Increasing the loading ratio of the phthalocyanine was associated with a decrease in the PDT efficiency even if the PDT-induced cell death remained significant. This can be attributed to the more important aggregation of phthalocyanine in the more loaded nanoparticles. **PEG**_**42**_**-*b*-PMLABe**_**73**_-based PEGylated nanoparticles were in comparison less efficient to induce cell death by PDT: **PEG**_**42**_**-*b*-PMLABe**_**73**_**[Pc1%]** showed 33% of cell death (Figure 5B), whereas more loaded **PEG**_**42**_**-*b*-PMLABe**_**73**_**[Pc5%]** and **PEG**_**42**_**-*b*-PMLABe**_**73**_**[Pc10%]** nanoparticles did not induce cell death. It was evident that the photodynamic efficiency of non-PEGylated Pc-loaded nanoparticles is higher than that of PEGylated counterparts. In addition, the lack of PDT effect of empty nanoparticles was verified as a negative control and Figure S11 demonstrated the total absence of killing with or without laser irradiation at 50 μg mL^–1^. Finally, a dose–response study of nonencapsulated **ZnPc(SO**_**2**_**Prop)8** incubated with MCF-7 cells was also performed using Pc concentrations corresponding to the concentrations in the nanoformulations (0.5 μg mL^–1^ for [Pc1%], 2.5 μg mL^–1^ for [Pc5%] and 5 μg mL^–1^ for [Pc10%]). Figure S12 demonstrates that no PDT effect was observed, although there is a decrease in the number of living cells with increasing Pc concentration.

**Figure 6:**
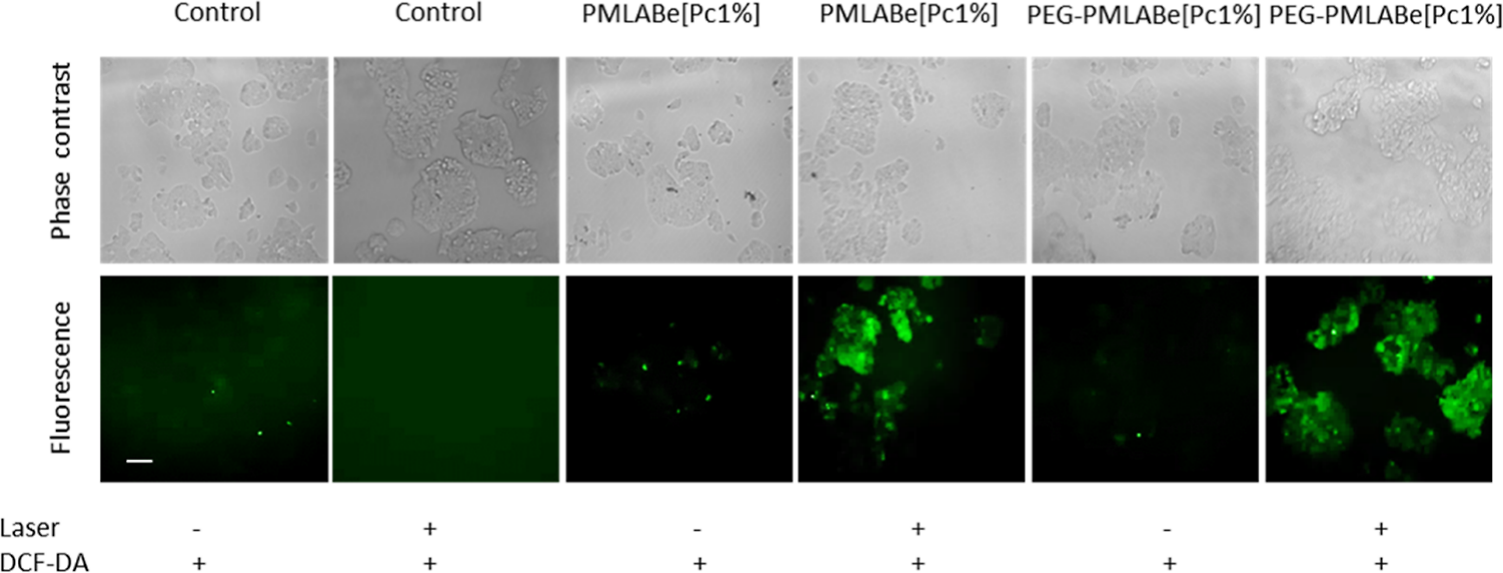
Detection of intracellular ROS generation using the DCFDA assay in MCF-7 cells treated with 50 μg mL^–1^ of nanoparticles for 24 h and then cells were irradiated or not with continuous laser for 20 min at 650 nm (11.25 J cm^–2^). Scale bar: 20 μm.

A qualitative experiment has been conducted to assess the generation of intracellular ROS as a consequence of PDT was confirmed in MCF-7 cells using a DCFDA assay. Cells were incubated with 50 μg.mL^–1^ of **PMLABe**_**73**_**[Pc1%]** and **PEG**_**42**_**-*b*-PMLABe**_**73**_**[Pc1%]** for 24 h and then exposed or not to laser irradiation as previously described. Results showed that nanoparticle-treated cells that were not exposed to laser irradiation showed no or low green fluorescent. Exposure to laser irradiation induced an increase in the green fluorescence intensity (Figure 6), which confirmed that ROS generation and following cell death is due to nanoparticles-induced PDT effect. It is important to emphasize that the difference in PDT effect measured 2 days after laser excitation (91% for **PMLABe**_**73**_**[Pc1%]** and 33% for **PEG–PMLABe**_**73**_**[Pc1%]**) should not be precisely connected to this imaging experiment of ROS production that is done immediately after laser irradiation and is only qualitative, not quantitative.

Despite the better encapsulation and less phthalocyanine aggregation observed in the PEGylated nanoparticles, it is the more hydrophobic **PMLABe**_**73**_-based nanoparticles that exhibited the best PDT effect. One may rather attribute these differences in photodynamic efficiency to the more efficient cellular internalization of the of **PMLABe**_**73**_-based nanomaterials, while those of the PEGylated nanoparticles is slower due to the shedding effect of the PEG moieties, a phenomenon known as the PEG dilemma.^30^ Unfortunately, the **ZnPc(SO**_**2**_**Prop)8**-loaded nanoparticles are not sufficiently luminescent to confirm this hypothesis by confocal microscopy, but we plan to investigate this deeper in future studies.

## 4 Conclusions

The encapsulation of the octapropylsulfonyl-substituted **ZnPc(SO**_**2**_**Prop)8** in two different PMLABe-based polymeric materials, a hydrophobic non-PEGylated (**PMLABe**_**73**_) and an amphiphilic PEGylated derivative (**PEG**_**42**_**-*b*-PMLABe**_**73**_), has been successfully achieved. The effect of the loading ratio and of the (co)polymer type has been investigated, showing that encapsulation is more efficient into PEGylated **PEG**_**42**_**-*b*-PMLABe**_**73**_-based nanoparticles, which also slightly decreases the aggregation of the phthalocyanine inside the nanoparticles. However, the photodynamic efficiency was much more efficient for the hydrophobic **PMLABe**_**73**_-based nanoparticles, which again confirms the relevance of the wide use of PEGylation in biomedical applications.

## Supporting Information Available

Characterization spectra of the polymeric materials and of **ZnPc(SO**_**2**_**Prop)8**; spectrum for the preparation of the calibration curve for E.E. calculation; materials and methods for UV–visible and fluorescence measurements; UV–visible spectra of nanoparticles in water; fluorescence spectra of disrupted nanoparticles; dark toxicity and PDT effect of empty **PMLABe**_**73**_ and **PEG**_**42**_-***b*-PMLABe**_**73**_ nanoparticles; and dark toxicity and PDT effect of nonencapsulated **ZnPc(SO**_**2**_**Prop)8** (PDF)
