# Supplementary material for: Nanoencapsulation of a Far-Red Absorbing Phthalocyanine into Poly(benzylmalate) Biopolymers and Modulation of Their Photodynamic Efficiency
Source: Biomacromolecules. 2024 May 16;25(6):3261–70. doi: 10.1021/acs.biomac.3c01382 (PMC11170942; doi:10.1021/acs.biomac.3c01382)
Supplement: Supplementary file 1 — bm3c01382_si_001.pdf [file bm3c01382_si_001.pdf]

Supplementary information for

Nano-encapsulation of a far-red absorbing phthalocyanine into poly(benzylmalate) biopolymers and modulation of their photodynamic efficiency

*Zeynel Şahin,[a†] Emel Önal,[b†] Lamiaa M. A. Ali,[c,d†] Denis Durand,[c] Atefeh Emami,[e] Marie Touré[c], Umit İşci,[a] Magali Gary-Bobo[*c], Sandrine Cammas-Marion[*f,g] and Fabienne Dumoulin[*e]*

[a] Marmara University, Faculty of Technology, Department of Metallurgical & Materials Engineering, 34722 Istanbul, Türkiye

[b] Doğuş University, Faculty of Engineering, Ümraniye, 34775 Istanbul, Türkiye

[c] IBMM, Univ Montpellier, CNRS, ENSCM, 34093 Montpellier, France

[d] Department of Biochemistry Medical Research Institute, University of Alexandria, 21561 Alexandria, Egypt

[e] Acıbadem Mehmet Ali Aydınlar University, Faculty of Engineering and Natural Sciences, Biomedical Engineering Department, Ataşehir, 34752 Istanbul, Türkiye.

[f] Univ Rennes, ENSCR, INSA Rennes, CNRS, ISCR (Institut des Sciences Chimiques de Rennes) – UMR 6226, F-35000 Rennes, France

[g] INSERM, INRAE, Univ Rennes, Institut NUMECAN (Nutrition Metabolisms and Cancer), U 1317, F-35000 Rennes, France

[†] Equal contributions

Correspondance to : magali.gary-bobo@inserm.fr, sandrine.marion.1@ensc-rennes.fr and fabienne.dumoulin@acibadem.edu.tr

[†] Equal contributions

Contents

Characterization spectra for polymeric materials **PMLABe$_{73}$** and **PEG$_{42}$-*b*-PMLABe$_{73}$**... 3

Figure S1. ^{1}H NMR spectrum (400 MHz, CD_3COCD_3) of **PMLABe$_{73}$** and PEG$_{42}$-*b*-PMLABe$_{73}$.......... 3

Figure S2. ^{1}H NMR spectrum (400 MHz, CD_3COCD_3) of **PEG$_{42}$-*b*-PMLABe$_{73}$**.............. 3

Figure S3. SEC chromatogram (THF, 40°C, polystyrene standards, RI detector) of **PMLABe$_{73}$**........... 4

Figure S4. SEC chromatogram (THF, 40°C, polystyrene standards, RI detector) of **PEG$_{42}$-*b*-PMLABe$_{73}$**........... 4

Characterization spectra for **ZnPc(SO$_2$Prop)8**........... 5

Figure S5. FT-IR spectrum of **ZnPc(SO$_2$Prop)8**........... 5

Figure S6. ^{1}H NMR spectrum of **ZnPc(SO$_2$Prop)8**........... 5

Figure S7. MALDI-MS spectrum of **ZnPc(SO$_2$Prop)8**

Preparation of calibration curve for E.E........... 7

Figure S8. A: UV-vis spectra of **ZnPc(SO$_2$Prop)8** in a 9/1 THF/water mixture. B: Calibration curve........... 7

Materials and Methods for UV-visible and Fluorescence measurements........... 8

UV-visible spectra of nanoparticles in water........... 9

Figure S9. UV-vis spectra (water) of **ZnPc(SO$_2$Prop)8**-loaded **PMLABe$_{73}$** nanoparticles (red) and **ZnPc(SO$_2$Prop)8**-loaded **PEG$_{42}$-*b*-PMLABe$_{73}$** nanoparticles (blue) with different loading ratios: 1 wt% (dot), 5 wt% (dash) and 10 wt% (line). Phthalocyanine concentrations: 2-8 µM........... 9

Fluorescence spectra of disrupted nanoparticles........... 10

Figure S10. Absorbance (line), fluorescence emission (dashes) and excitation (dots) spectra of **ZnPc(SO$_2$Prop)8**-loaded **PMLABe$_{73}$** disrupted nanoparticles (red), of **ZnPc(SO$_2$Prop)8**-loaded **PEG$_{42}$-*b*-PMLABe$_{73}$** disrupted nanoparticles (blue) and of **ZnPc(SO$_2$Prop)8** (black), with different loading ratios........... 10

Figure S11. Dark toxicity and PDT effect of empty **PMLABe$_{73}$** and **PEG$_{42}$-*b*-PMLABe$_{73}$** nanoparticles........... 11

Figure S12. Dark toxicity and PDT effect of non-encapsulated **ZnPc(SO$_2$Prop)8**........... 11

References........... 12

Characterization spectra for polymeric materials

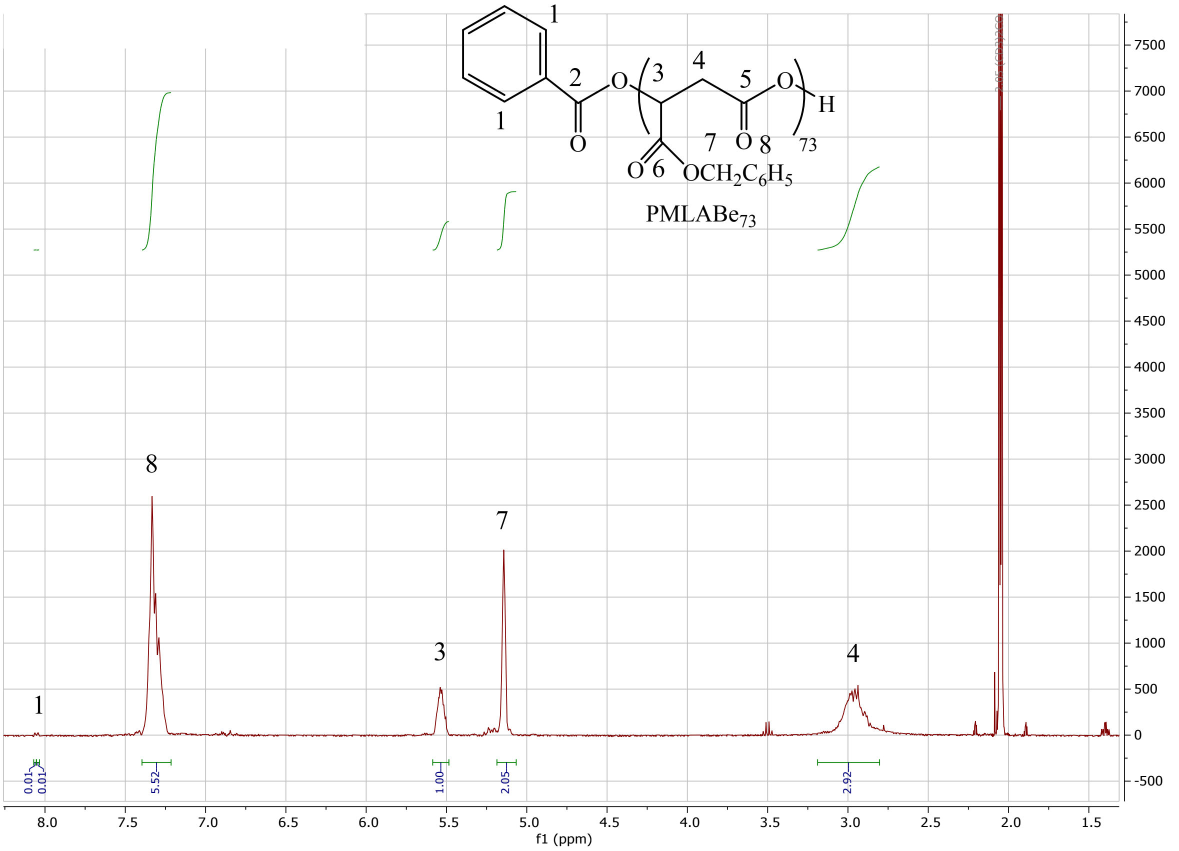

Figure S1. ^{1}H NMR spectrum (400 MHz, CD_3COCD_3) of **PMLABe**$_{\mathbf{73}}$ and PEG$_{42}$-*b*-PMLABe$_{73}$.

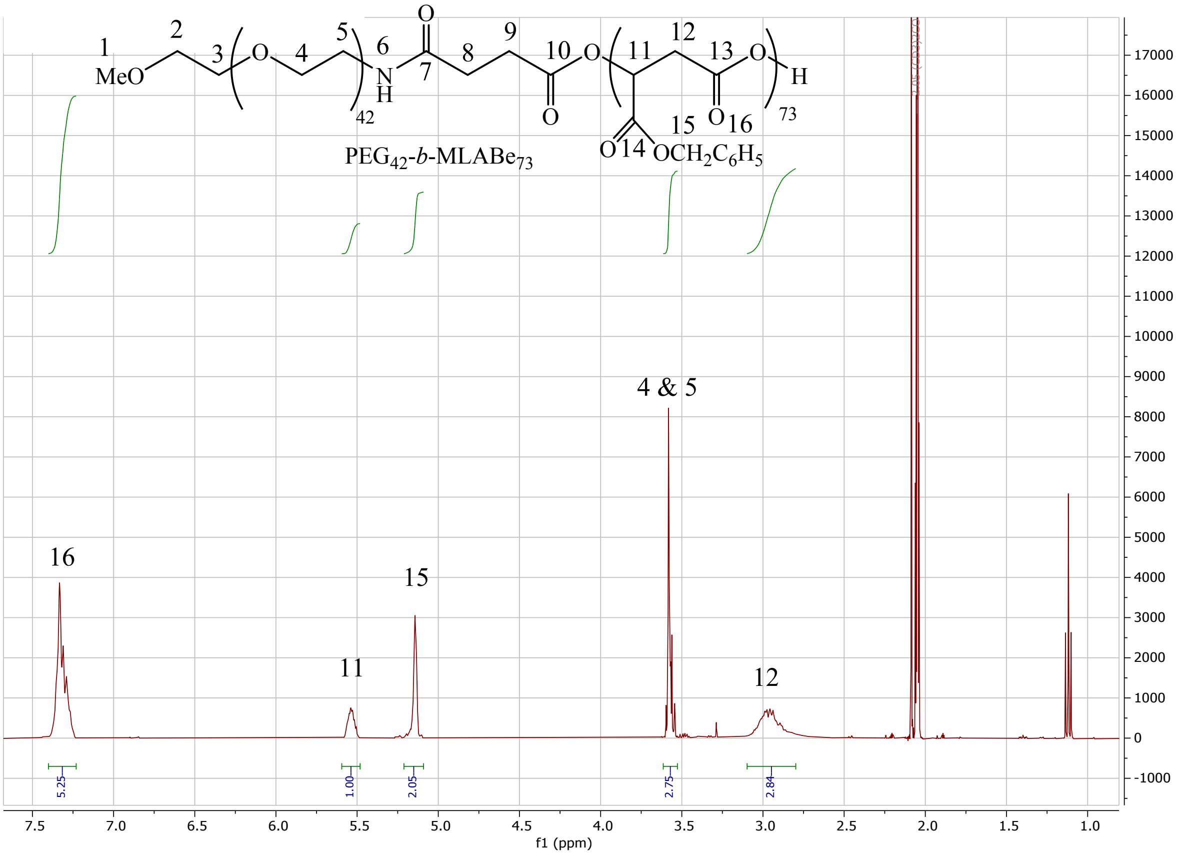

Figure S2. ^{1}H NMR spectrum (400 MHz, CD_3COCD_3) of **PEG**$_{\mathbf{42}}$**-*b*-PMLABe**$_{\mathbf{73}}$.

.

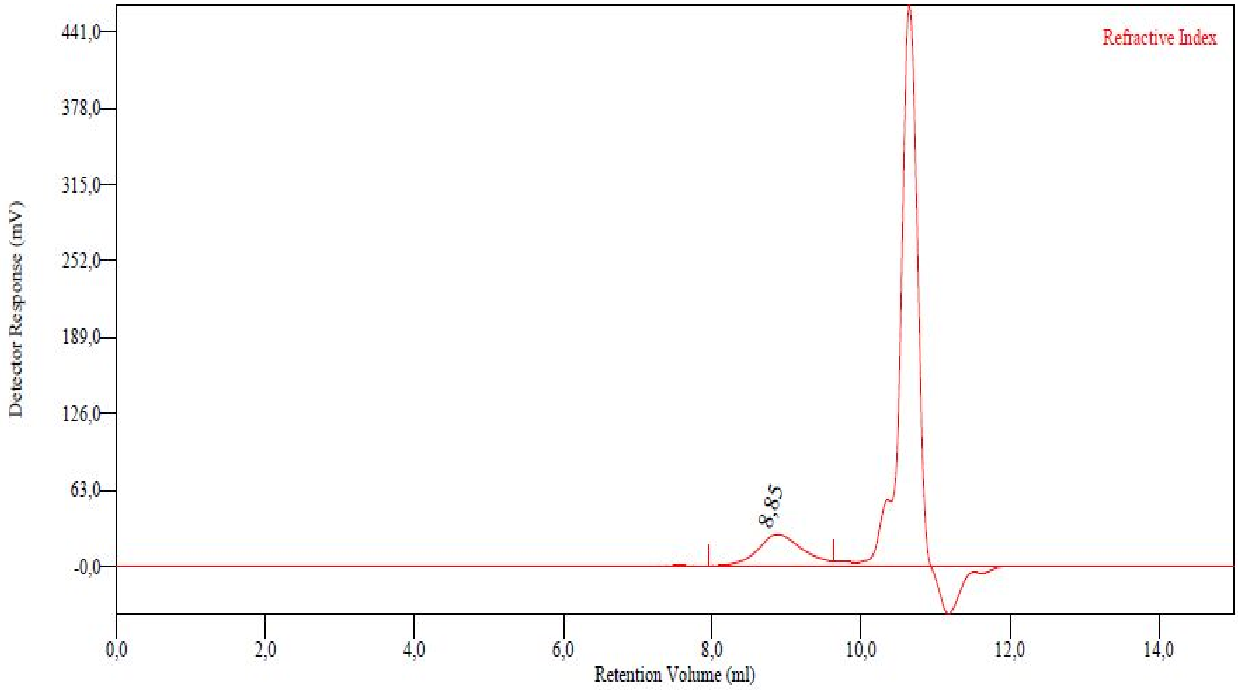

Figure S3. SEC chromatogram (THF, 40°C, polystyrene standards, RI detector) of **PMLABe$_{73}$**.

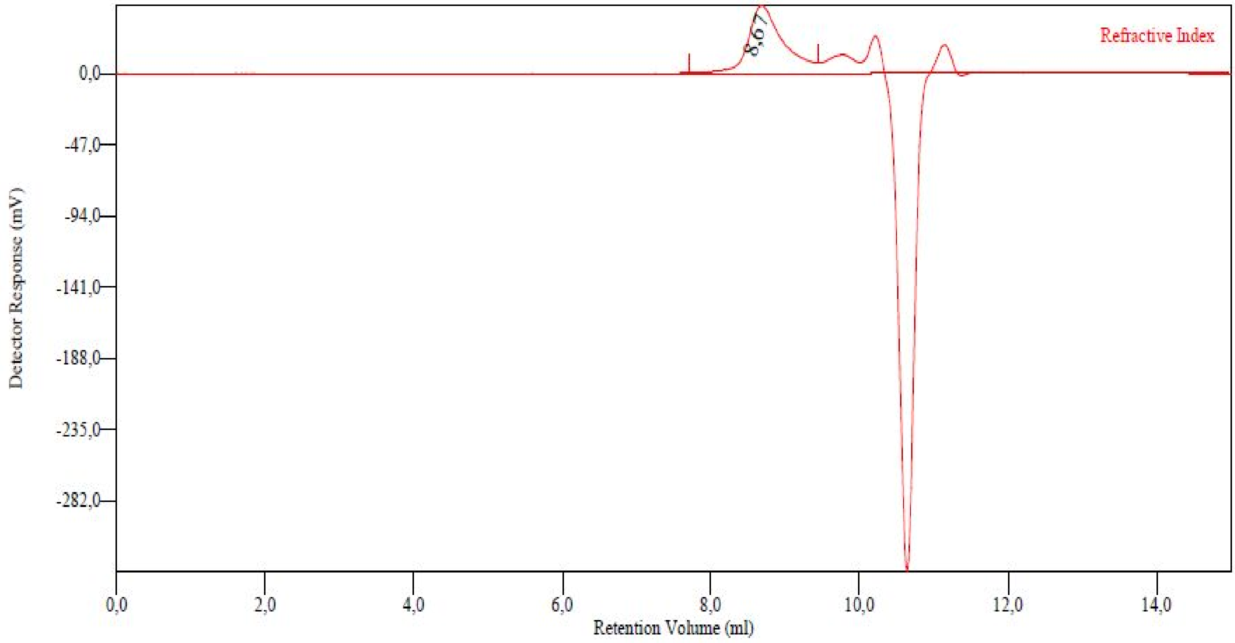

Figure S4. SEC chromatogram (THF, 40°C, polystyrene standards, RI detector) of **PEG$_{42}$-*b*-PMLABe$_{73}$**.

Characterization spectra for ZnPc(SO_2Prop)8

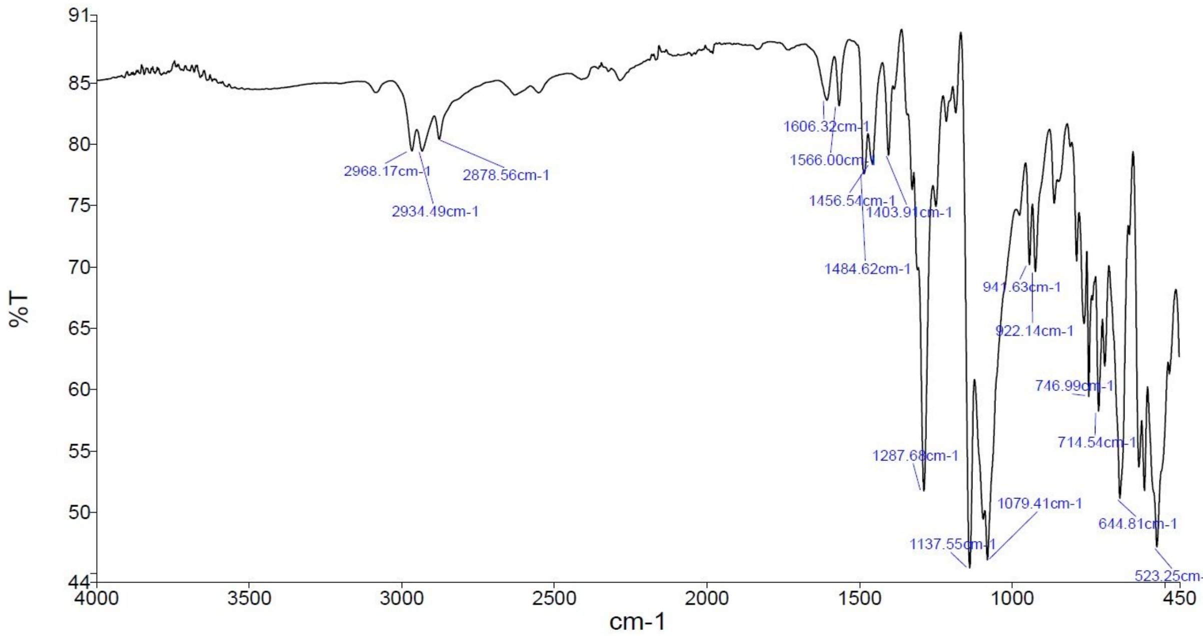

Figure S5. FT-IR spectrum of **ZnPc(SO_2Prop)8**

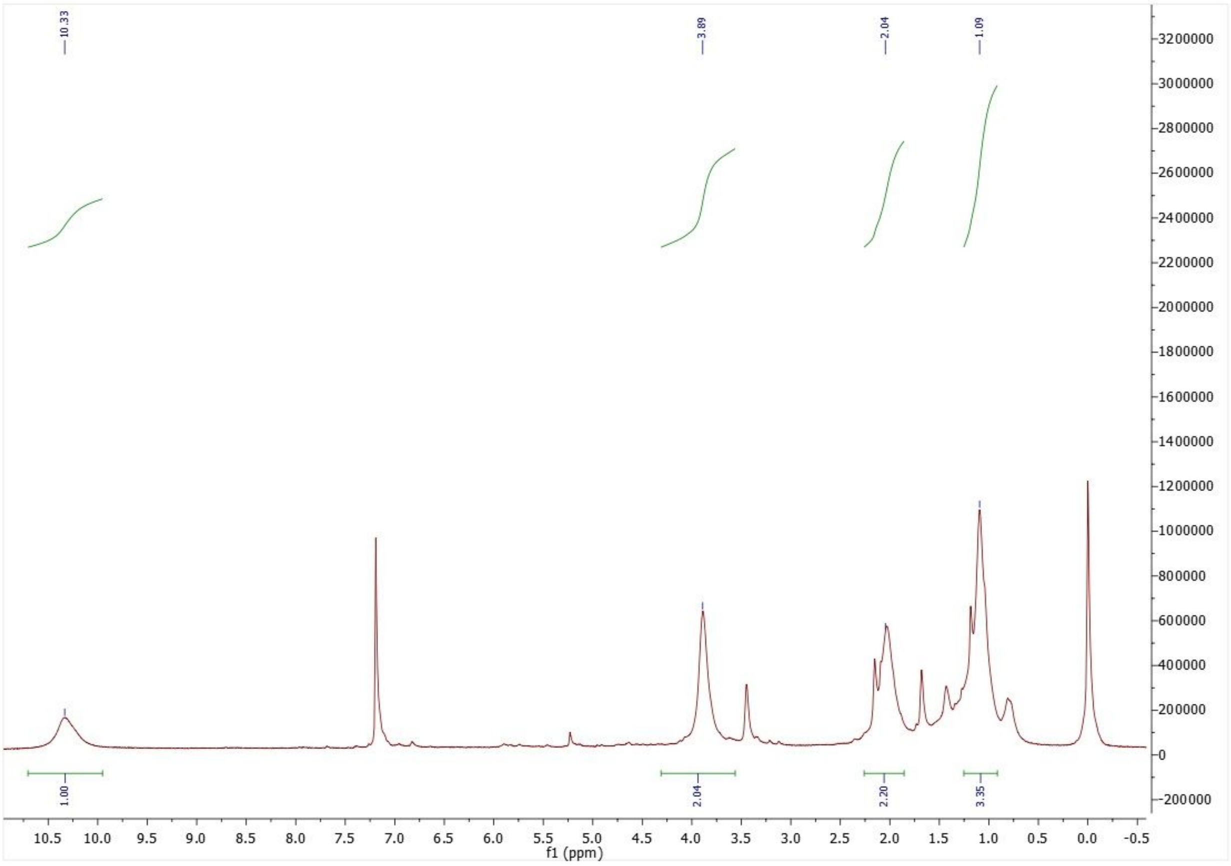

Figure S6. ^{1}H NMR spectrum of **ZnPc(SO_2Prop)8** ($CDCl_3$)

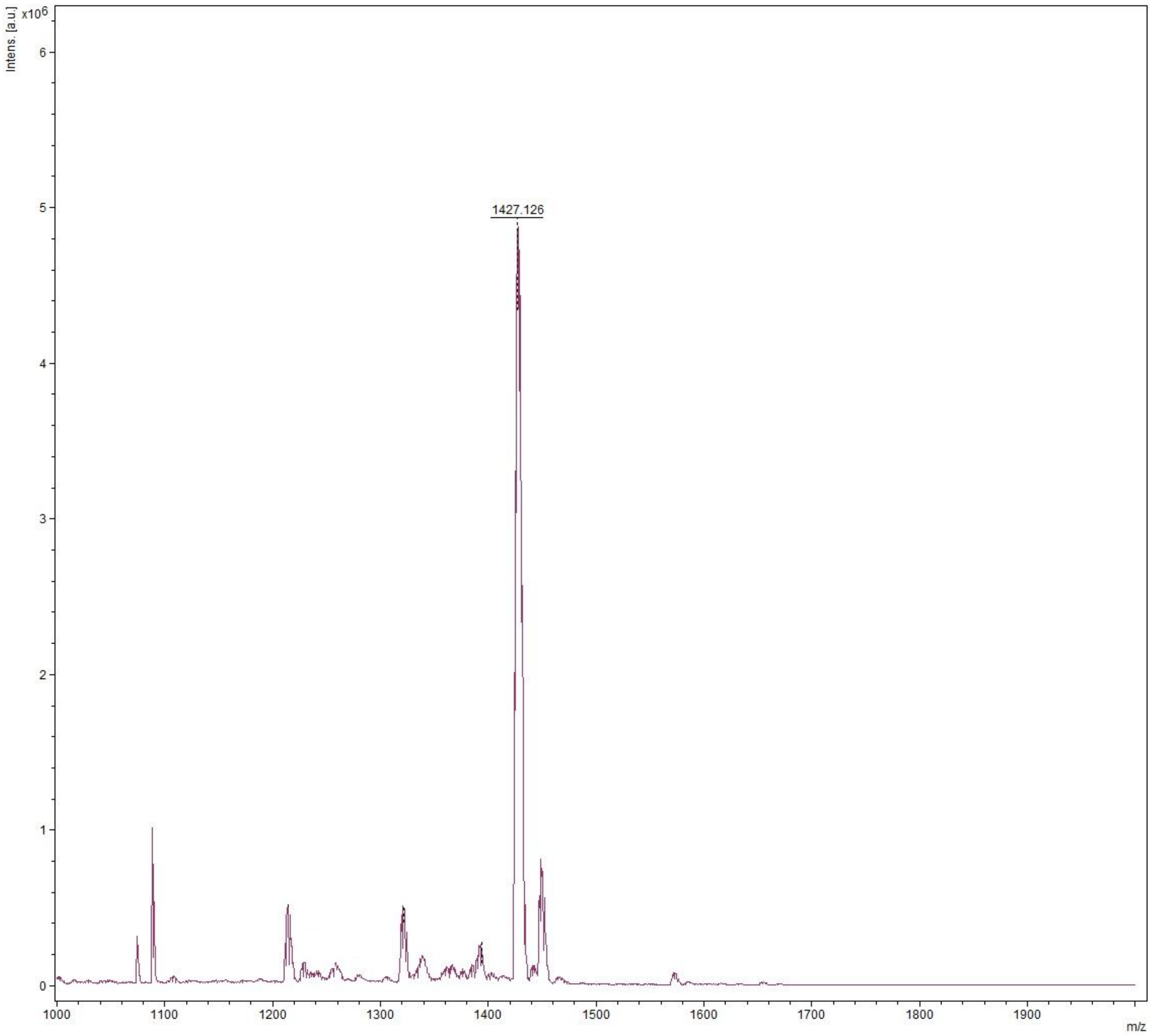

Figure S7. MALDI-MS spectrum of **ZnPc(SO_2Prop)8**

Preparation of calibration curve for E.E.

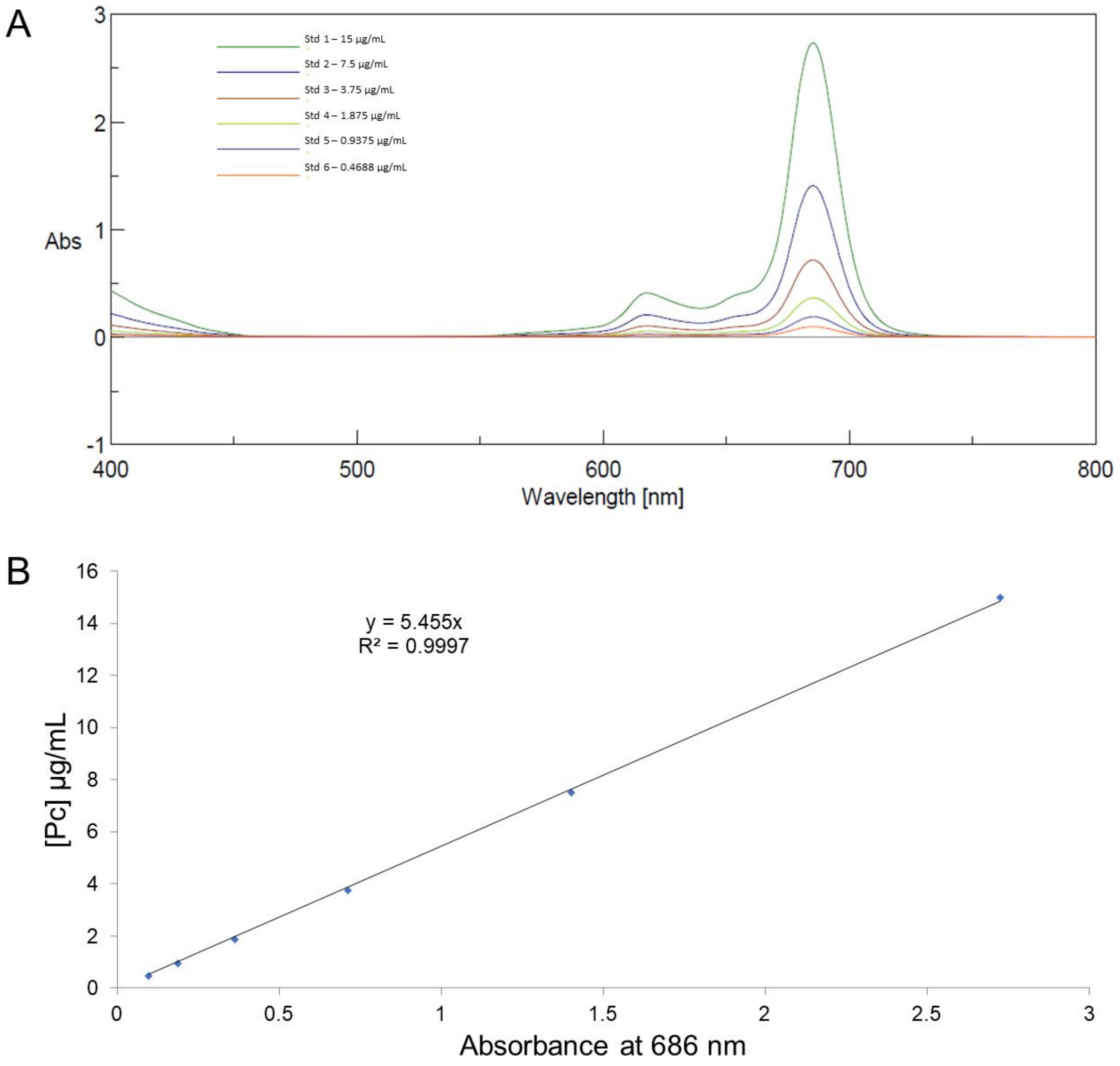

Figure S8. A: UV-vis spectra of **ZnPc(SO_2Prop)8** in a 9/1 THF/water mixture. B: Calibration curve.

Materials and Methods for UV-visible and Fluorescence measurements

Ground state electronic absorption

Absorption spectra were recorded at room temperature on a Schimadzu 2600 UV-vis spectrometer using a 1 cm path length quartz cuvette between maximum range 300 and 1100 nm in THF. Solutions were freshly prepared in spectrophotometric grade solvents. Molar extinction coefficients (ε) were determined by measurement of the absorption solutions of differing concentration for each compound, followed by determination of the slope.

Fluorescence

Steady-state fluorescence excitation and emission spectra in THF were recorded by using a Varian Cary Eclipse spectrofluorometer using 10 mm path length cuvettes at room temperature.

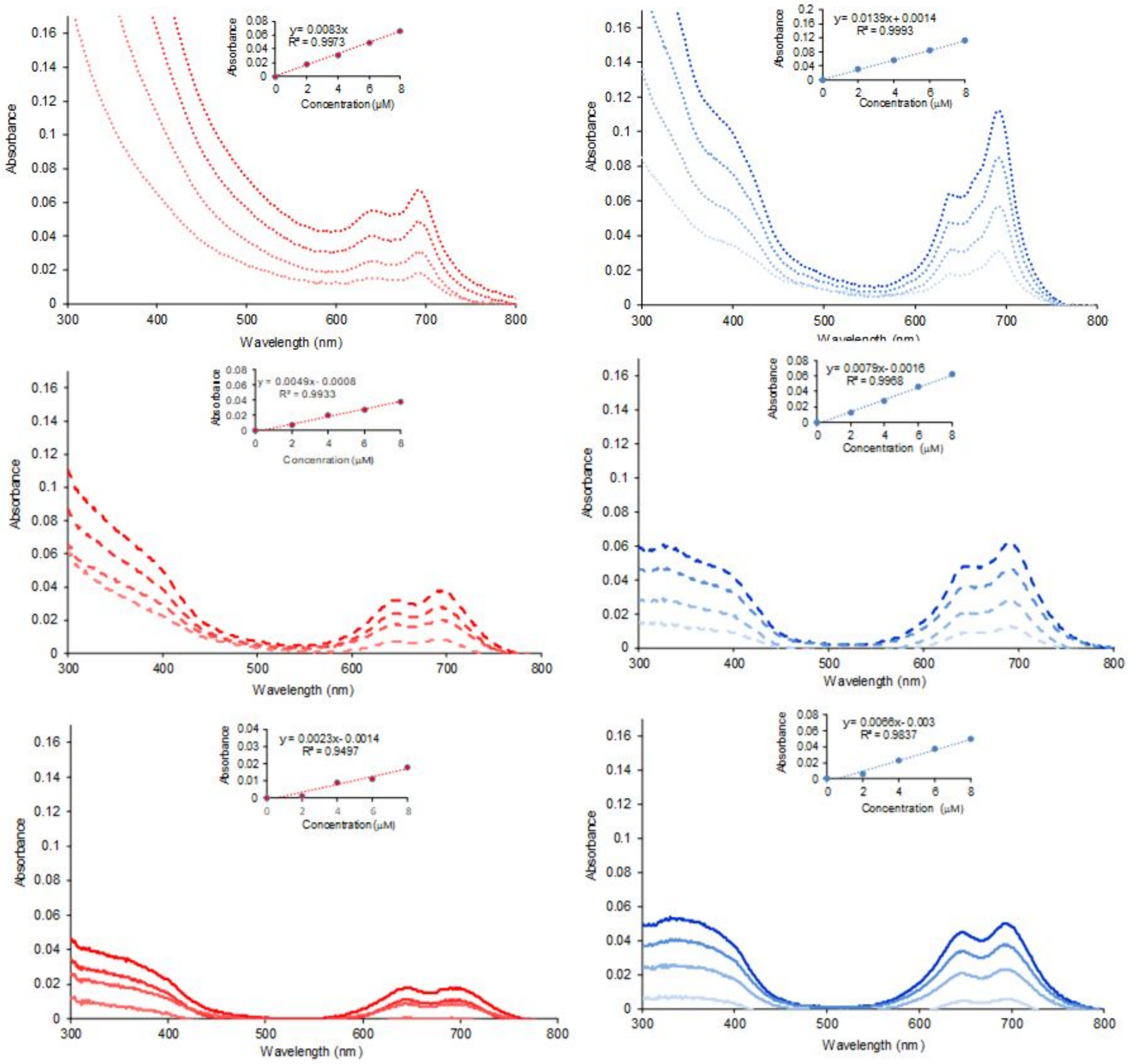

Figure S9. UV-vis spectra (water) of **ZnPc(SO_2Prop)8**-loaded **PMLABe$_{73}$** nanoparticles (red) and **ZnPc(SO_2Prop)8**-loaded **PEG$_{42}$-*b*-PMLABe$_{73}$** nanoparticles (blue) with different loading ratios: 1 wt% (dot), 5 wt% (dash) and 10 wt% (line). Phthalocyanine concentrations: 2-8 µM.

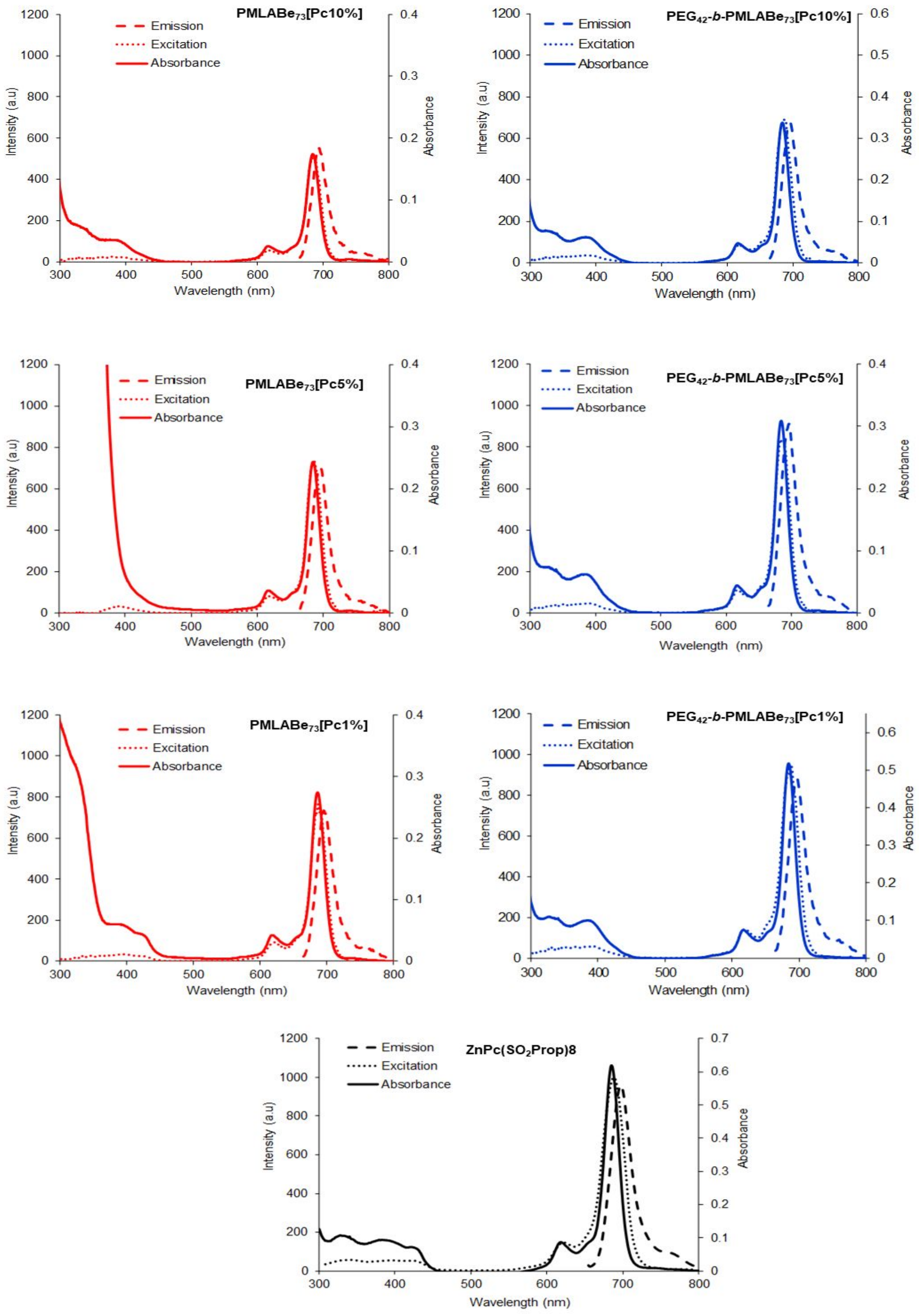

Figure S10. Absorbance (line), fluorescence emission (dashes) and excitation (dots) spectra (in THF) of **ZnPc(SO_2Prop)8**-loaded **PMLABe$_{73}$** disrupted nanoparticles (red), of **ZnPc(SO_2Prop)8**-loaded **PEG$_{42}$-*b*-PMLABe$_{73}$** disrupted nanoparticles (blue) and of **ZnPc(SO_2Prop)8** (black), with different loading ratios.

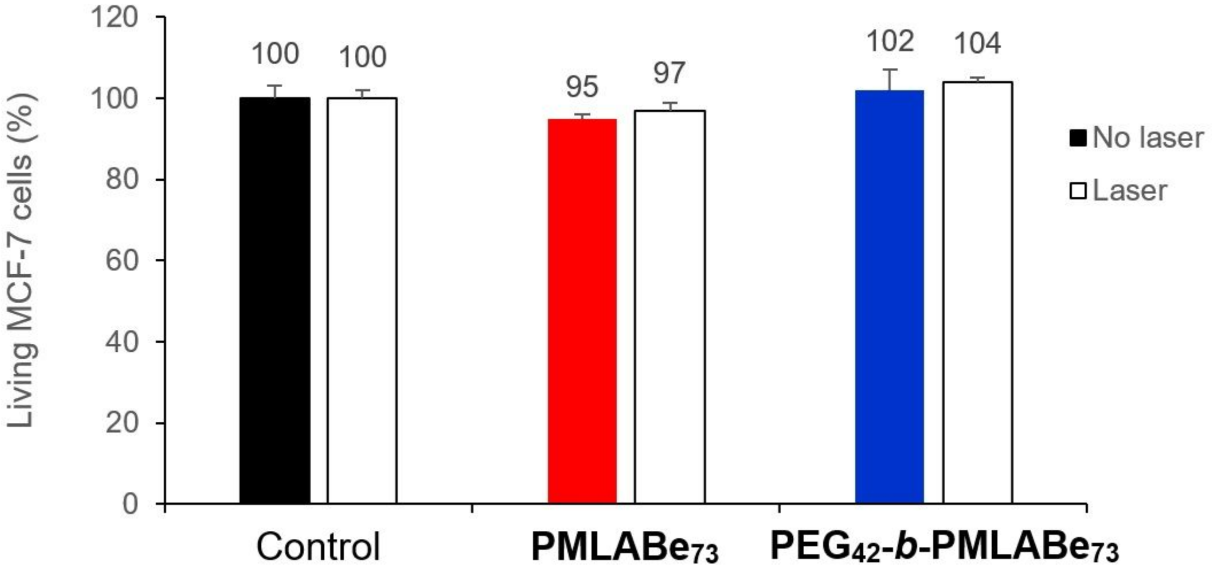

Figure S11. Study of PDT effect of empty **PMLABe$_{73}$** and **PEG$_{42}$-*b*-PMLABe$_{73}$** nanoparticles, incubated 24 h at 50 µg.mL^{-1} with MCF-7 cells. Cells were irradiated with continuous laser for 20 min at 650 nm. Data are presented as (mean ± SD), n=3.

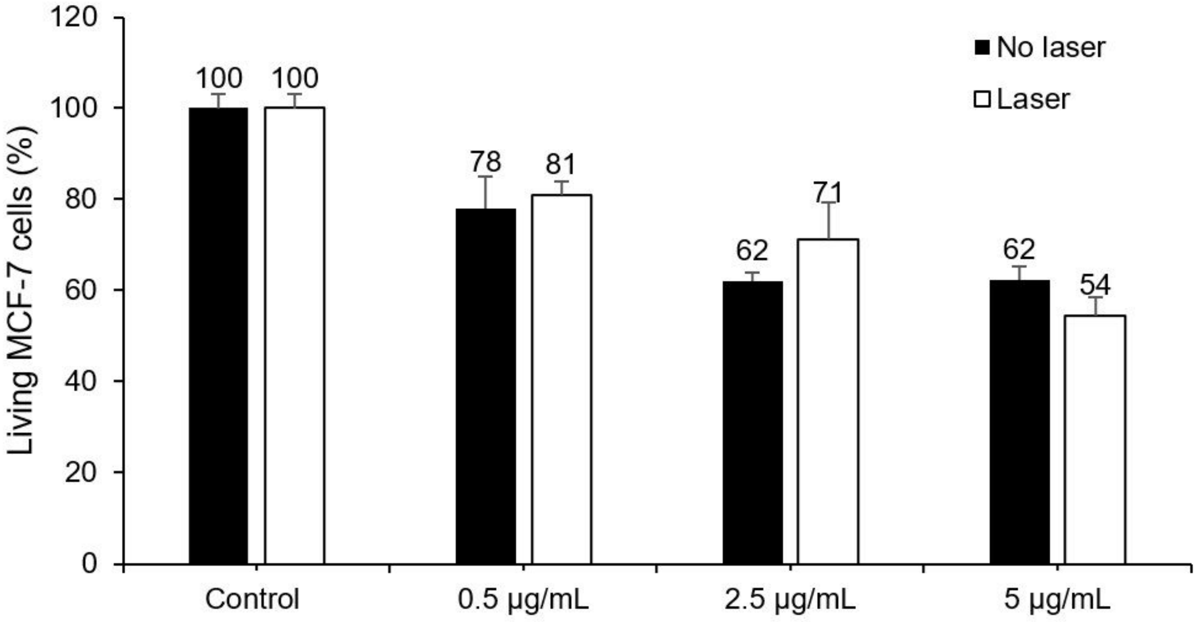

Figure S12. Study of PDT effect of non-encapsulated **ZnPc(SO$_2$Prop)8** on MCF-7 cells treated with 0.5 µg/mL, 2.5 µg/mL and 5 µg/mL for 24 h. Cells were irradiated with continuous laser for 20 min at 650 nm. Data are presented as (mean ± SD), n=3.

References

[1] Williams, A. T. R.; Winfield, S. A.; Miller, J. N. Relative Fluorescence Quantum Yields Using a Computer-Controlled Luminescence Spectrometer. *Analyst*, **1983**, 108 (1290), 1067–1071.

[2] Ogunsipe, A.; Maree, D.; Nyokong, T. Solvent effects on the photochemical and fluorescence properties of zinc phthalocyanine derivatives. *Journal of Molecular Structure*. **2003**, 650, 131-140.
